# Supplementary material for: Post‐metaphase correction of aberrant kinetochore‐microtubule attachments in mammalian eggs
Source: EMBO Rep. 2019 Jul 10;20(8):e47905. doi: 10.15252/embr.201947905 (PMC6680117; doi:10.15252/embr.201947905)
Supplement: Supplementary file 1 — Expanded View Figures PDF [file EMBR-20-e47905-s001.pdf]

Expanded View Figures

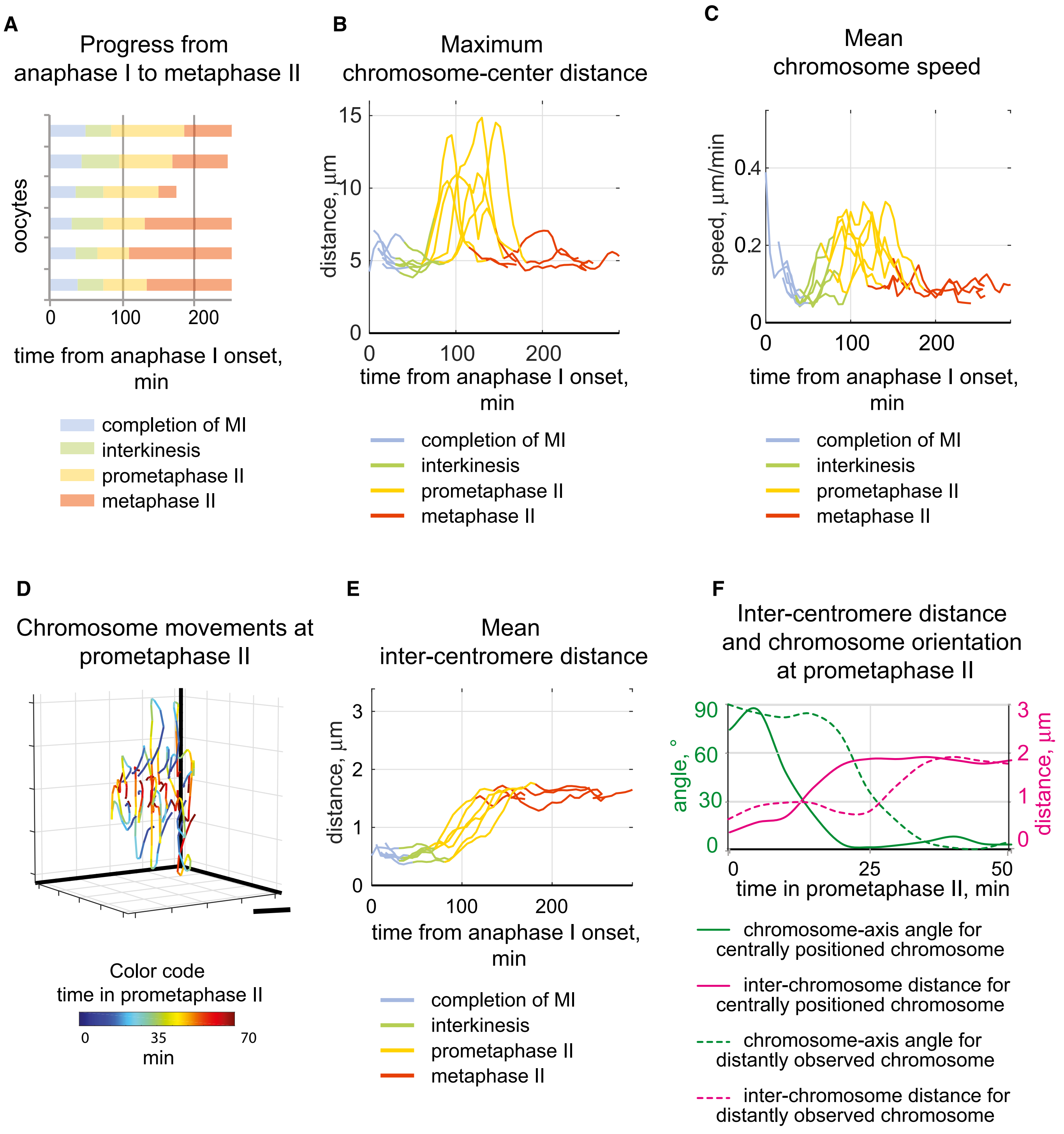

Figure EV1.

◀

Figure EV1. Anaphase I to metaphase II arrest parameters in oocytes.

Chromosome parameters were calculated for 6 oocytes undergoing the anaphase I to metaphase II transition. Colour of the line segments in A, B, C and E corresponds to the stage, as indicated below the graphs.

- A The duration of four stages between anaphase I and metaphase II. Horizontal axes show time after anaphase I onset (min) for six analysed oocytes. The individual stages are colour-coded, as indicated below the chart.
- B Separation of most distantly located chromosomes from the centre in analysed oocytes. Maximal values up to 15 μm are observed at prometaphase II (yellow segments).
- C Mean chromosome speed in individual oocytes increases at prometaphase II (yellow segments) and then decreases at metaphase II (red segments).
- D The 3D tracks of individual chromosomes at prometaphase II stage are shown for a representative oocyte, demonstrating that chromosomes move parallel to each other. The colour code shows the time after beginning of the prometaphase II stage as indicated by the colour bar. Scale bar, 5 μm.
- E The mean inter-centromere distance in analysed oocytes increases to 1.6 ± 0.2 μm (mean ± SD) during prometaphase II stage (yellow segments).
- F Inter-centromere stretching at the prometaphase II stage is coordinated with achieving the correct orientation parallel to the spindle axis. Changes in the chromosome orientation (green lines) and inter-centromere distance (magenta lines) during the prometaphase II stage are shown for a centrally positioned chromosome (solid lines), and a distantly observed chromosome (dashed lines). The chromosome-axis angle values are shown on the left vertical axes, and inter-centromere distance values are laid out on the right vertical axes. The time passed after beginning of the prometaphase II stage is shown in minutes on the horizontal axis.

 © 2019 The Authors

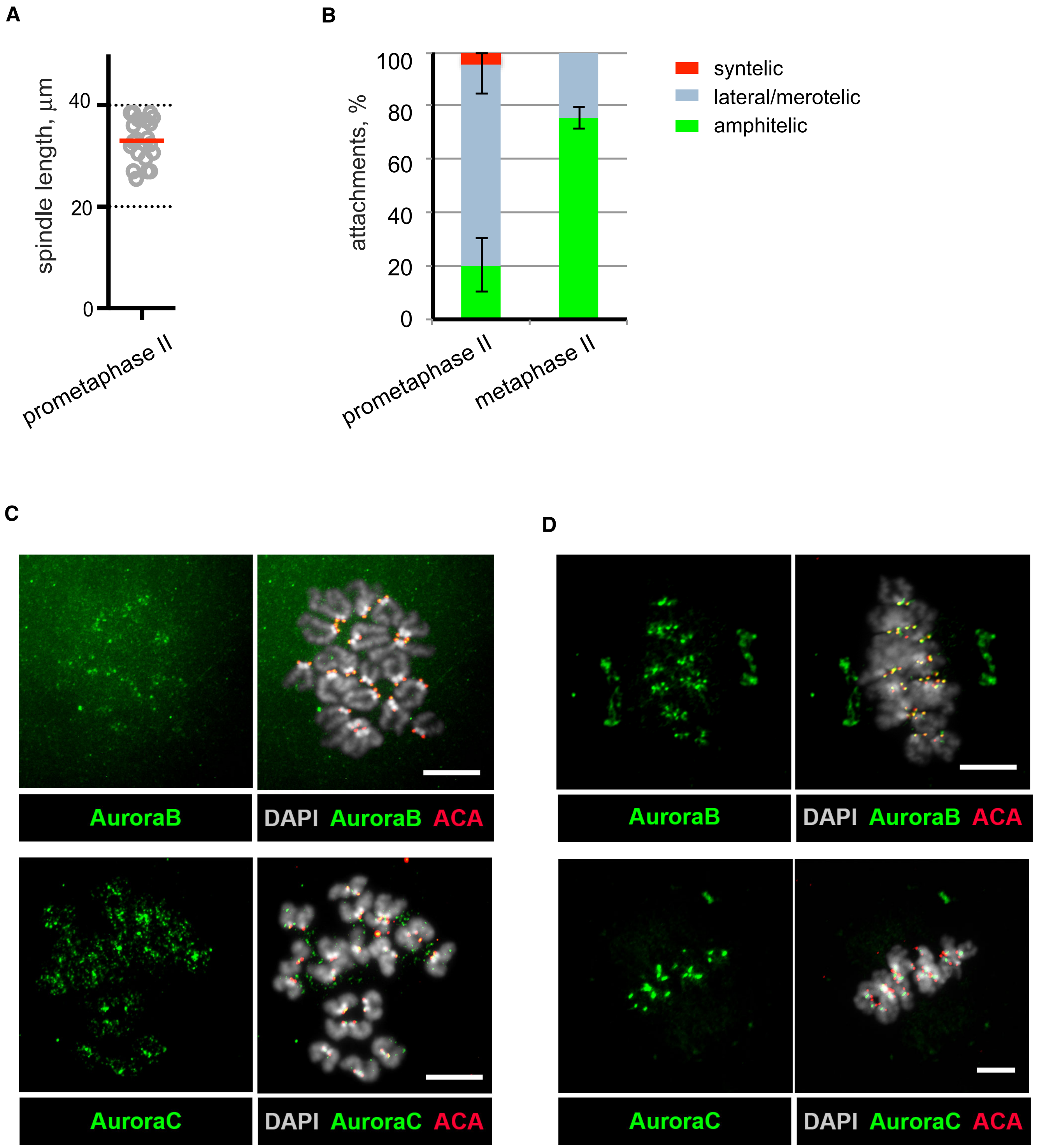

Figure EV2. Chromosome attachments to the spindle and Aurora B/Aurora C localization at prometaphase II stage.

A Spindle length at prometaphase II was 33 ± 4.3 µm (Mean ± SD, n = 20).

B Percentage of syntelic (red), amphitelic (green) and merotelic/lateral (grey) attachments in MII oocytes at prometaphase II and CSF-dependent metaphase II arrest (mean ± SD, n = 156 from 8 oocytes at the prometaphase and 98 from 5 oocytes at the metaphase stages).

C, D Aurora B (green, top) and Aurora C (green, bottom) were visualized on chromosomes at prometaphase II (C) and at CSF-dependent metaphase II arrest (D). Centromeres were labelled by ACA (red), and chromatin stained by DAPI (grey). Scale bars, 10 µm.

© 2019 The Authors

Figure EV3. Mean chromosome-axis angle, chromosome speed and inter-centromere distance from metaphase II to anaphase II.

A Maximum distances to spindle axis (plotted on the horizontal axis) and spindle equatorial plane (plotted on the vertical axis) are stable after release from CSF-dependent metaphase arrest until anaphase II onset. The analysed distances are indicated on the scheme by black arrowed lines; spindle axis is shown by solid line and spindle equator by dashed line; chromosomes are red with green centromeres. The maximum distances were calculated at each time point for 14 oocytes released from CSF-dependent metaphase arrest, and each oocyte is shown in a unique colour (*n* = 14).

B, C Mean chromosome-axis angle before anaphase II onset (plotted on the vertical axis) is low as a vast majority of the chromosomes are oriented almost parallel to the spindle axis with a mean chromosome-axis angle equal to 6 ± 2° (mean ± SD).

D, E Mean chromosome speed (on the vertical axis) is low before anaphase II onset, with a mean value of 0.1 ± 0.03 μm/min (mean ± SD).

F, G Mean inter-centromere distance (on the vertical axis) is relatively constant for all chromosomes before anaphase II onset. The mean inter-chromosome distance is 1.7 ± 0.2 μm (mean ± SD).

Mean parameters were calculated for all chromosomes for each time point, representing the variability observed in individual oocytes from metaphase II to anaphase II (B, D, F), or for each chromosome for the last 30 min before the anaphase onset, representing the variability between chromosomes (C, E, G). In B, D and F, time is shown in minutes on the horizontal axis relative to anaphase II onset, and each oocyte is represented by a unique colour (*n* = 14). In C, E and G, individual oocytes are arranged along the horizontal axis (*n* = 14), grey circles represent individual chromosomes (*n* = 20 in each oocyte); red lines indicate the median values for each oocyte.

 © 2019 The Authors

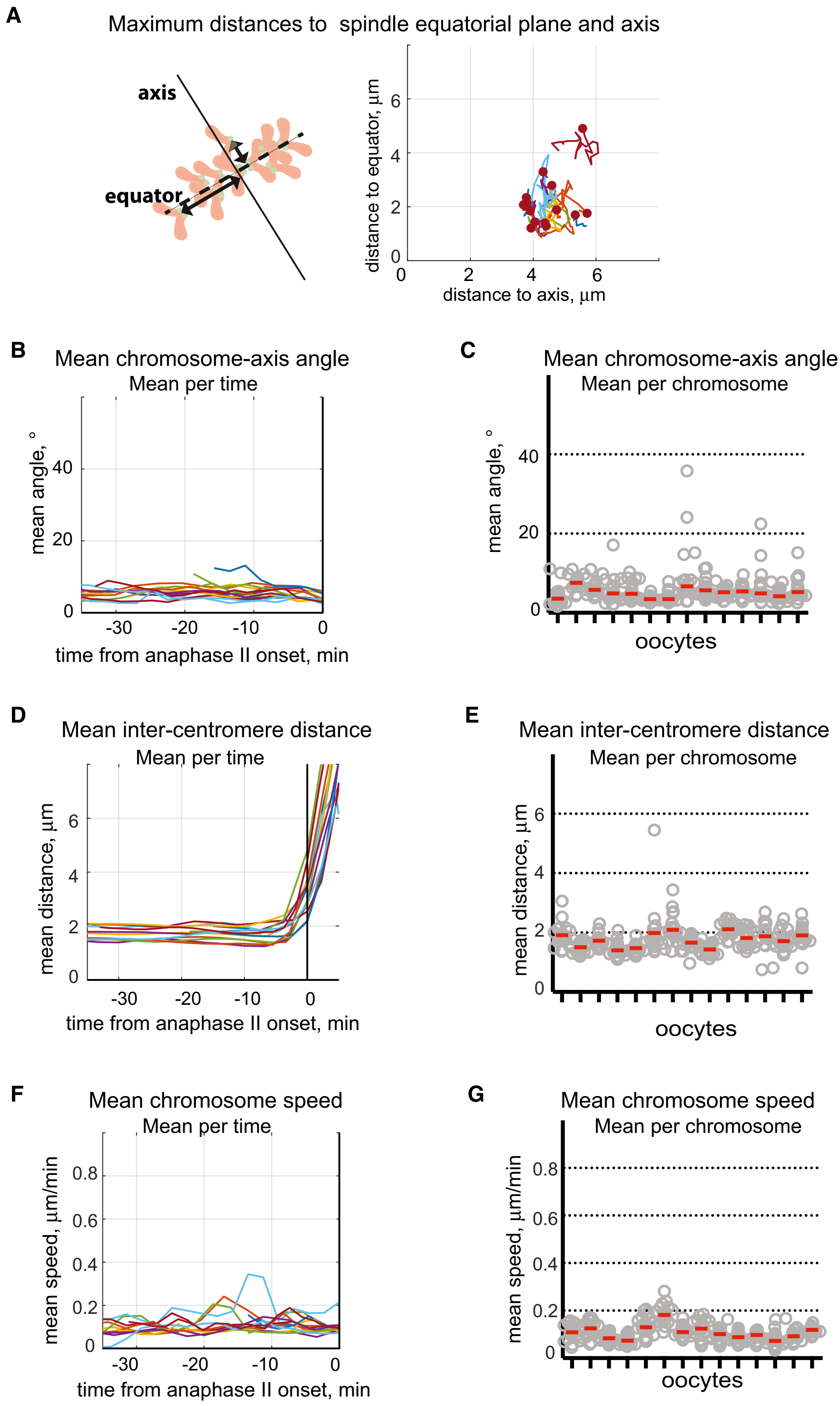
A
Maximum distances to spindle equatorial plane and axis
axis
equator
distance to equator, µm
distance to axis, µm
B
Mean chromosome-axis angle
Mean per time
mean angle, °
time from anaphase II onset, min
C
Mean chromosome-axis angle
Mean per chromosome
mean angle, °
oocytes
D
Mean inter-centromere distance
Mean per time
mean distance, µm
time from anaphase II onset, min
E
Mean inter-centromere distance
Mean per chromosome
mean distance, µm
oocytes
F
Mean chromosome speed
Mean per time
mean speed, µm/min
time from anaphase II onset, min
G
Mean chromosome speed
Mean per chromosome
mean speed, µm/min
oocytes

Figure EV3.

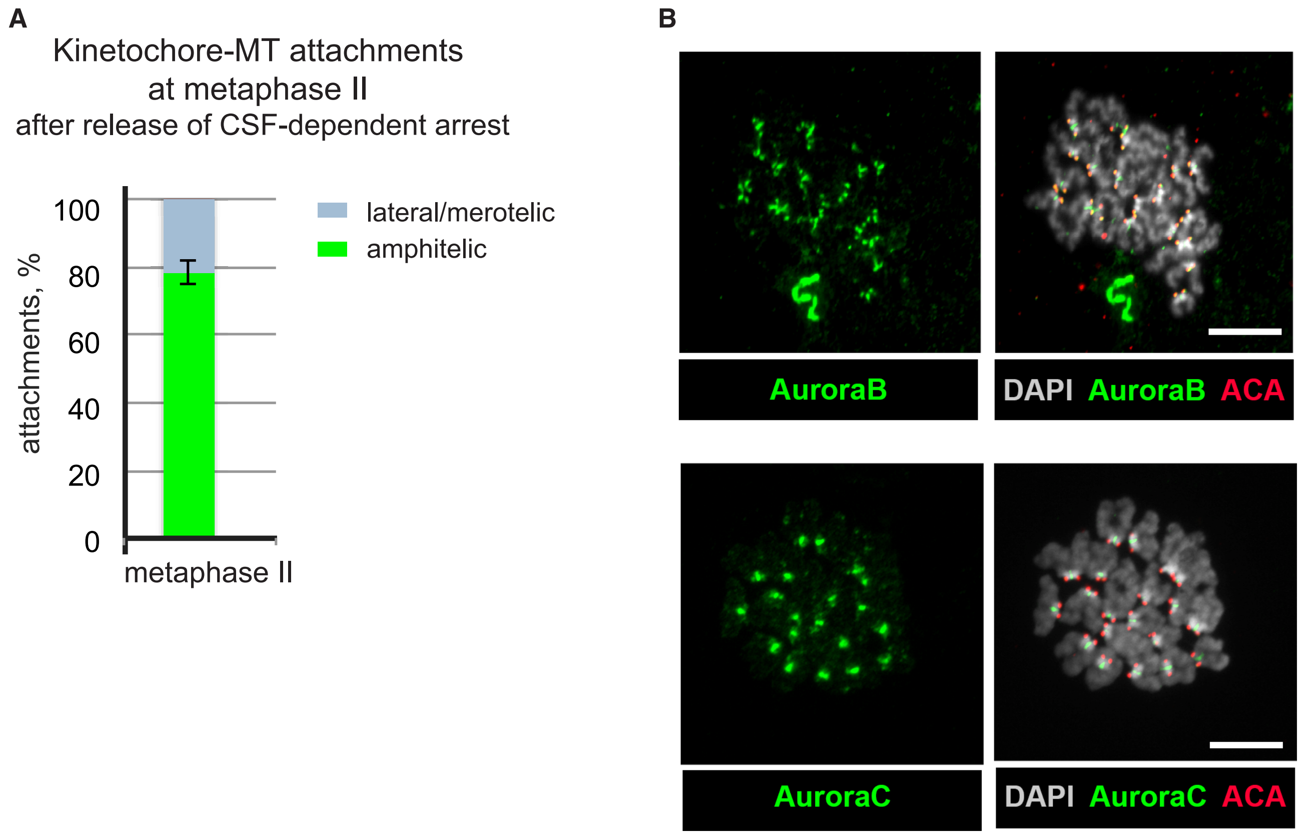

Figure EV4. Chromosome attachments to the spindle and Aurora B/Aurora C localization at metaphase II stage.

A Percentage of amphitelic (green) and merotelic/lateral (grey) attachments at metaphase II stage after release from CSF-dependent metaphase arrest (mean ± SD, n = 98 from 5 oocytes).

B Aurora B (green, top) and Aurora C (green, bottom) were visualized on chromosomes after release from CSF-dependent metaphase II arrest. Centromeres were labelled by ACA (red) and chromatin stained by DAPI (grey). Scale bars, 10 µm.

 © 2019 The Authors

Figure EV5. Second meiotic division with lagging chromatids.

A Lagging chromatids do not cause a delay in anaphase II onset. Anaphase starts at 70 ± 25 min after activation for oocytes with a normal segregation pattern (green diamonds) and at 68 ± 20 min for oocytes with lagging chromatids (blue diamonds) (mean ± SD, n = 14 and 10, respectively). Red lines indicate the mean values.

B Speed for laggard-producing chromosomes in MII oocytes (blue lines for 12 laggard-producing chromosomes with equational segregation and dark blue line for one chromosome segregating with non-disjunction). Black dotted line indicates the mean speed of the normally segregated chromosomes in all analysed oocytes (n = 10). The time is shown in minutes on the horizontal axis relative to anaphase II onset.

C–E Chromosome speed (C), chromosome-axis angle (D) and inter-chromosome distance (E) averaged for the last 30 min before anaphase II onset for each chromosome in oocytes displaying laggards (n = 10). Individual oocytes are distributed along the horizontal axis. Grey circles indicate the values displayed by normally segregating chromosomes, and blue dots represent the laggard-producing chromosomes with balanced chromatid separation; dark blue dot represents the chromosome segregating with chromatid non-disjunction. Red lines show the median values in each oocyte. Mean speeds of the laggard-producing oocytes (C) are similar to the speed of normally segregating chromosomes (P = 0.5, two-way ANOVA). Laggard-producing chromosomes demonstrate elevated chromosome-axis angle and reduced inter-centromere distance comparing to the normally segregating chromosomes (D and E, $P < 0.001$, two-way ANOVA).

F Inter-centromere distance is negatively correlated with the chromosome angle to the spindle axis for laggard-producing chromosomes (Pearson's correlation coefficient $r = -0.6$; P = 0.03). The inter-centromere distance for laggard-producing chromosomes is shown on the horizontal axis, and the angle between chromosome and spindle axis is shown on the vertical axis. Blue dots represent the average values for laggard-producing chromosomes with balanced chromatid separation; dark blue dot represents the average value for the chromosome where segregating results in chromatid non-disjunction.

G Changes in the inter-centromere distance and chromosome orientation in a representative laggard-producing chromosome. Arrows point to the periods where a decrease in inter-chromosome distance (magenta line) coincides with an increase in chromosome orientation with respect to the spindle axis (green line). Chromosome-axis angle values are shown on the left vertical axes, while inter-centromere distance values are laid out on the right vertical axes. The time is shown in minutes on the horizontal axis relative to the anaphase II onset.

H Variability of the inter-centromere distances at the metaphase II stage is similar between normally segregating chromosomes and laggard-producing chromosomes. The standard deviation of inter-centromere distance is shown on the vertical axis. Blue dots represent the laggard-producing chromosomes with balanced chromatid separation; dark blue dot represents the chromosome segregating with chromatid non-disjunction, grey circles indicate the values displayed by normally segregating chromosomes from the same oocytes, and red lines indicate the median values (n = 187 for normally segregating chromosomes and 13 for laggard-producing chromosomes).

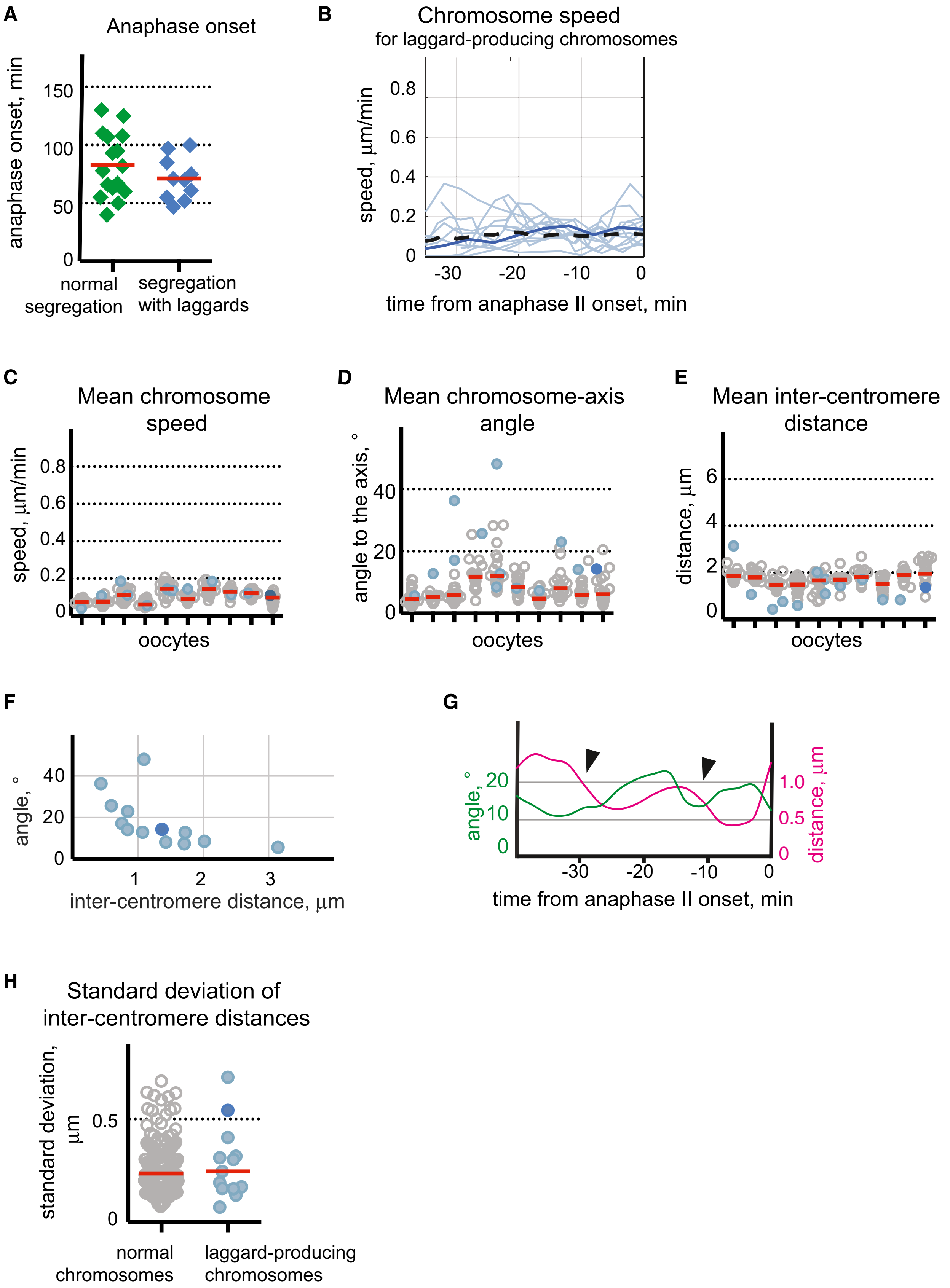

Figure EV5.

 © 2019 The Authors
