# Supplementary material for: Post‐metaphase correction of aberrant kinetochore‐microtubule attachments in mammalian eggs
Source: EMBO Rep. 2019 Jul 10;20(8):e47905. doi: 10.15252/embr.201947905 (PMC6680117; doi:10.15252/embr.201947905)
Supplement: Supplementary file 2 — Movie EV1 [file EMBR-20-e47905-s002.zip › Movie_EV1.docx]

**Movie EV1.**

**Centromere and chromatin dynamics from anaphase I to the metaphase II arrest stage in a representative oocyte.** Maximum intensity z-projections images of EGFP-CENP-C (green, centromeres) and H2B-mCherry (red, chromatin) are shown. Time after anaphase I onset (hr:min:sec). Bar: 5 μm.
