# Supplementary material for: Post‐metaphase correction of aberrant kinetochore‐microtubule attachments in mammalian eggs
Source: EMBO Rep. 2019 Jul 10;20(8):e47905. doi: 10.15252/embr.201947905 (PMC6680117; doi:10.15252/embr.201947905)
Supplement: Supplementary file 4 — Movie EV3 [file EMBR-20-e47905-s004.zip › Movie_EV3.docx]

**Movie EV3.**

**Centromere and chromatin dynamics from metaphase to anaphase II in a representative oocyte displaying lagging chromatids at the anaphase II.** Blue arrow points to the position of a lagging centromere, showing a correct (equational) segregation pattern. Maximum intensity z-projections images of EGFP-CENP-C (green, centromeres) and H2B-mCherry (red, chromatin) are shown. Time relative to anaphase II onset (min:sec). Bar: 5 μm.
